# Prevalence of overweight and obesity among Chinese Yi nationality: a cross-sectional study

**DOI:** 10.1186/1471-2458-11-919

**Published:** 2011-12-13

**Authors:** Yun Gao, Xing-Wu Ran, Xiao-Hua Xie, Hua-Lin Lu, Tao Chen, Yan Ren, Yang Long, Hao-Ming Tian

**Affiliations:** 1 Department of Endocrinology and Metabolism, West China Hospital of Sichuan University, 37 GuoXue lane, Chengdu 610041, People's Republic of China; 2 Department of Endocrinology and Metabolism, The First People's Hospital of Liangshan Yi Nationality Autonomy District, Xichang City 615000, People's Republic of China; 3 Laboratory of Endocrinology and Metabolism, West China Hospital of Sichuan University, Chengdu 610041, People's Republic of China

## Abstract

**Background:**

Overweight and obesity are considered a serious health problem. There are little data on the prevalence of overweight and obesity among the Yi ethnic group in China. This study aimed to investigate the epidemiologic features of overweight/obesity among Chinese Yi nationality.

**Methods:**

A cross-sectional study, including 1255 subjects aged 20-75 years, was carried out in Liangshan Yi Autonomous Prefecture of Sichuan province from 2007 to 2008. Overweight/overall obesity was defined by World Health Organization (WHO) or the Working Group on Obesity in China.

**Results:**

Overall, the prevalence of overweight and obesity was 19.0% and 2.9%, respectively, based on the WHO definition, while it was 21.0% and 7.4%, respectively, according to the Working Group on Obesity in China, which is similar to data reported in the 2002 Chinese National Nutrition and Health Survey. Urban residents had a significantly higher prevalence of obesity (WHO criteria: 4.3% vs 1.7% *p *= 0.008; China criteria: 11.4% vs 3.7%, *p *< 0.001) and overweight (WHO criteria: 28.9% vs 8.9% *p *< 0.001; China criteria: 31.2% vs 10.4%, *p *< 0.001) than that in rural residents. Older age, a family history of obesity, higher income, drinking and urban residence were significantly associated with an increased risk of overweight/obesity.

**Conclusions:**

The prevalence of overweight/obesity in the Yi nationality is similar to that in Chinese adults 5 years ago. However, urban residents have a much higher prevalence of overweight/obesity than their rural counterparts. Lifestyle and diet patterns associated with socioeconomic status may explain the difference between urban and rural residents. The prevention of overweight/obesity among urban inhabitants deserves more attention in national health education programs.

## Background

Over the past several decades, the economic transition in China has provoked sweeping changes in lifestyle involving overconsumption of dietary fat and reduction in physical activity, which have contributed to an increase in body weight (BW) [1,2]. Several studies have shown that the prevalence of overweight and obesity is increasing to epidemic proportions at an alarming rate in the Chinese population [2,3]. This upsurge in obesity propels the growing prevalence of hypertension and type 2 diabetes, which substantially exacerbate the national economic burden [4].

Liangshan Yi Autonomous Prefecture within Sichuan province in southwestern China, of which a brief introduction is given in additional file 1, contains the largest Yi community in China. In recent years, the whole area has grown at an accelerated rate and people's livelihoods have improved constantly. Since epidemic status of obesity often coincides with economic growth and increased family income [5], we hypothesize that the prevalence of obesity is dramatically rising among the Yi nationality population, the minority in southwestern China. Moreover, as Yi people in urban areas often live together with the Han nationality, their dietary structure has been affected by the Han, whereas most residents in rural areas are only Yi nationality and they still retain their traditional dietary patterns. It is likely that there may be a significant rural-urban difference in the prevalence of overweight or obesity among the Yi ethnic group. However, no large-scale epidemiological data are available on the prevalence of overweight or obesity among the Yi nationality. Therefore, the objective of this cross-sectional study was to assess the prevalence and epidemiological characteristics of overweight and obesity among the Yi nationality in an autonomous prefecture.

## Methods

### Study population

We used a multistage, stratified sampling method to select a representative sample of persons aged 20 to 75 years in the general population. Subjects registered were permanent residents in Liangshan Yi Autonomous Prefecture within Sichuan province from July 2007 to May 2008. The sampling database was based on the 2000 National Bureau of Statistics of China. In the first stage, the whole autonomous prefecture was stratified into urban and rural areas. Urban areas comprised county-seats and Xichang City (the state capital). Two sample points from urban areas and 2 from rural areas were selected to be representative of the geographic and economic characteristics in their regions. In the second stage, 2 street districts or rural villages were randomly selected from each county-seat and rural township, respectively. Primary sampling units were street districts in urban areas and hamlets in rural sample points. In the third stage, one participant was selected from a randomly selected household in the primary sampling units. Simple random sampling methods were used at each stage. Using this method, a total of 1500 participants was randomly selected and was invited to participate; 1288 persons (571 men and 717 women) completed the study. The overall response rate was 85.9% (76.1% for men and 95.6% for women). After the exclusion of 33 persons for whom demographic information was missing, 1255 adults were included in the final analysis.

The study was approved by the ethics committee at West China Hospital, Sichuan University. Informed consent was obtained from each participant before data collection.

### Questionnaire and physical examinations

Data collection was conducted in examination centers at local health stations or in community clinics in the participants' residential area. Trained research staff administered a standard questionnaire to obtain information on demographic characteristics, personal and family history, and lifestyle risk factors [6]. The interview included questions related to the diagnosis and treatment of diabetes, hypertension, dyslipidemia, and cardiovascular events. Smokers included current smokers and ex-smokers; current smokers were those who smoked at least 1 cigarette per day lasting for at least 1 year, and ex-smokers were those who had regularly smoked in the past, but had not smoked for at least half a year. Information was obtained on the amount and type of alcohol that was consumed during the previous years, and alcohol drinking was defined as the consumption of at least 30 g of alcohol per week for 1 year or more. Regular leisure-time physical activity was defined as participation in moderate or vigorous activity for 30 min or more per day at least 3 days a week. Socioeconomic status, educational level, occupation, and income were also recorded. Blood pressure, BW, height, and waist circumference (WC) were measured with the use of standard methods, as described previously [7]. In brief, height and BW were measured without shoes and in light clothing after overnight fasting. Body mass index (BMI) was calculated by dividing BW (in kilograms) by height (in meters) squared. WC was measured at the horizontal plane between the inferior costal margin and the superior iliac crest on the midaxillary line.

The definition of overweight/obesity was based on the 1997 World Health Organization (WHO) criteria [8] or Working Group on Obesity in China [9]. According to the WHO criteria, obesity was defined as a BMI of at least 30 kg/m^2 ^and overweight was defined as 25 ≤ BMI < 30 kg/m^2^. According to the Working Group on Obesity in China, obesity was defined as a BMI of at least 28 kg/m^2 ^and overweight was defined as 24.0 ≤ BMI < 28 kg/m^2^.

All study investigators and staff members successfully completed a training program that familiarized them with both the aims of the study and the specific tools and methods used. At the training sessions, interviewers were given detailed instructions concerning the administration of the study questionnaire. Clinical staff members were trained to measure blood pressure and obtain anthropometric measurements and blood specimens according to a standard protocol [6].

### Laboratory measurements

Participants were instructed to maintain their usual physical activity and diet for at least 3 days prior to glucose testing. Overnight fasting blood samples were collected using vacuum tubes containing sodium fluoride, used to determine fasting plasma glucose and lipids. Subjects without a history of diabetes were administered an oral glucose tolerance test (OGTT) of 75 g glucose, whereas subjects with a previous diagnosis of diabetes were administered a standard meal test containing 80 g carbohydrates. Blood samples for glucose determination were collected at 30 and 120 min after either the OGTT or standard meal test. Plasma glucose (PG) was measured with the use of a hexokinase enzymatic method, and serum cholesterol and triglyceride (TG) levels were assessed enzymatically with the use of commercially available reagents, at the clinical biochemical laboratory in the First People's Hospital of Liangshan Yi Nationality Autonomy District, Xichang City. The laboratory has successfully completed a standardization and certification program.

### Statistical analysis

All analyses were performed using the Statistical Package for Social Sciences (SPSS for Windows, version 16.0; Chicago, IL). Age and sex standardization was performed by using the direct method with the 2000 China census data. We used the P-P plot to test the normality of the numerical variables. Data are presented as means ± SD, medians (interquartile range) or frequencies (number of cases). TG and PG were log-transformed because of skewed distributions. The significant differences of univariate comparisons between 2 groups were assessed by Student t tests or *χ*^2 ^tests. Multiple logistic regression analysis was performed to analyze the association of demographic factors and lifestyle with the odds of overweight/obesity.

## Results

Characteristics of the study participants based on urban-rural categories are presented in Table 1. Urban inhabitants had more drinkers, a higher education and income, a higher BMI, WC, systolic blood pressure (SBP), and diastolic blood pressure (DBP), and higher levels of PG, total cholesterol (TC), TG, high-density lipoprotein cholesterol (HDL-C), and low-density lipoprotein cholesterol (LDL-C) compared with their rural counterparts. Furthermore, urban residents were more likely to participate in leisure-time physical activity and less likely to smoke cigarettes than rural residents. No significant difference was found in age between urban and rural residents. Table 2 displays the sex-specific clinical characteristics. There were no differences in mean age, BMI and the levels of 2-h PG, TC, HDL-C, and LDL-C between male and female participants. There was a higher prevalence of smoking, drinking, and a higher education and income, higher WC, SBP, and DBP, and higher levels of fasting plasma glucose, 0.5-h PG, and TG in men than in women (Tables 1 and 2).

**Table 1:** Characteristics of study participants according to urban-rural categories

Characteristics	Urban residents (n = 621)	Rural residents (n = 634)	p value
Sex (F/M)	376/245	332/302	

Age	44.6 ± 14.7	45.1 ± 14.2	NS

Smokers (%, n)			

Current smokers	23.0(143)	38.0(241)	< 0.001

Ex-smokers	10.0(62)	1.7(11)	

Drinkers (%, n)	38.6(240)	30.3(192)	< 0.001

Regular leisure-time physical activity	63.5(393)	10.4(66)	< 0.001

Education (%, n)			

College or above	36.7(228)	3.0(19)	< 0.001

Middle school	37.7(234)	12.5(79)	

Primary school or below	25.6(159)	84.5(536)	

Family annual income (%, n)			

≥ 10000 yuan	58.8(365)	5.8(37)	< 0.001

< 10000 yuan	41.2(256)	94.2(597)	

Body mass index (kg/m2)	24.0 ± 3.5	21.1 ± 3.1	< 0.001

Waist circumference (cm)	84.3 ± 11.2	76.3 ± 8.5	< 0.001

Family history of obesity (%, n)	17.6(109)	3.2(20)	< 0.001

Systolic blood pressure (mmHg)	117.8 ± 18.8	109.8 ± 16.5	< 0.001

Diastolic blood pressure (mmHg)	80.3 ± 12.6	74.2 ± 10.9	< 0.001

fasting plasma glucose (mmol/L)	5.3 ± 1.7	4.9 ± 1.0	< 0.001

0.5-hr plasma glucose (mmol/L)	8.6(7.1, 10.1)	7.3(6.1, 8.7)	< 0.001

2-hr plasma glucose (mmol/L)	6.6(5.6, 8.8)	5.3(4.4, 6.5)	< 0.001

TC(mmol/L)	4.5 ± 1.0	4.1 ± 0.9	< 0.001

TG(mmol/L)	1.34(0.93, 2.05)	1.26(0.89,1.78)	0.019

HDL-C(mmol/L)	1.3 ± 0.3	1.1 ± 0.2	< 0.001

LDL-C(mmol/L)	2.6 ± 0.7	2.4 ± 0.6	< 0.001

Data were expressed as means ± SD, median (interquartile range) and percentage (number of cases) in normally distributed continuous, abnormally distributed continuous and categorical variables, respectively. Log-transformed values for abnormally distributed continuous variables were used for analysis. Between-groups comparisons were conducted using Student t tests or *χ*2 tests. NS indicates not significant. TC: total cholesterol; TG: triglyceride; HDL-C: high-density lipoprotein cholesterol; LDL-C: low-density lipoprotein cholesterol

**Table 2:** Characteristics of study participants according to sex categories

Characteristics	Men (n = 547)	Women (n = 708)	p value
Age	45.6 ± 14.6	44.3 ± 14.3	NS

Smokers			

Current smokers	60.3(330)	7.6(54)	< 0.001

Ex-smokers	10.8(59)	2.0(14)	

Drinkers	59.4(325)	15.1(107)	< 0.001

Regular leisure-time physical activity	39.9(218)	34.1(241)	0.038

Education			

College or above	24.7(135)	15.8(112)	< 0.001

Middle school	28.9(158)	21.9(155)	

Primary school or below	46.4(255)	62.3(441)	

Family annual income(%, n)			

≥ 10000 yuan	36.7(201)	28.4(201)	0.002

< 10000 yuan	63.3(346)	71.6(507)	

Body mass index (kg/m2)	22.5 ± 3.5	22.6 ± 3.7	NS

Waist circumference(cm)	81.9 ± 11.0	79.0 ± 10.2	< 0.001

Family history of obesity (%, n)	9.0(49)	11.3(80)	NS

Systolic blood pressure(mmHg)	115.6 ± 17.0	112.3 ± 18.9	0.001

Diastolic blood pressure(mmHg)	78.6 ± 11.7	76.2 ± 12.4	< 0.001

fasting plasma glucose (mmol/L)	5.2 ± 1.5	5.0 ± 1.2	0.005

0.5-hr plasma glucose (mmol/L)	8.3(7.1, 9.8)	7.6(6.3, 9.0)	< 0.001

2-hr plasma glucose (mmol/L)	6.0(4.7, 7.8)	6.0(4.9, 7.4)	NS

TC(mmol/L)	4.3 ± 1.0	4.3 ± 1.6	NS

TG(mmol/L)	1.34(0.91, 1.98)	1.29(0.90,1.79)	0.023

HDL-C(mmol/L)	1.2 ± 0.3	1.2 ± 0.3	NS

LDL-C(mmol/L)	2.5 ± 0.7	2.5 ± 0.7	NS

Data were expressed as means ± SD, median (interquartile range) and percentage (number of cases) in normally distributed continuous, abnormally distributed continuous and categorical variables, respectively. Log-transformed values for abnormally distributed continuous variables were used for analysis. Between-groups comparisons were conducted using Student t tests or *χ*2 tests. NS indicates not significant. TC: total cholesterol; TG: triglyceride; HDL-C: high-density lipoprotein cholesterol; LDL-C: low-density lipoprotein cholesterol

**Figure 1:**
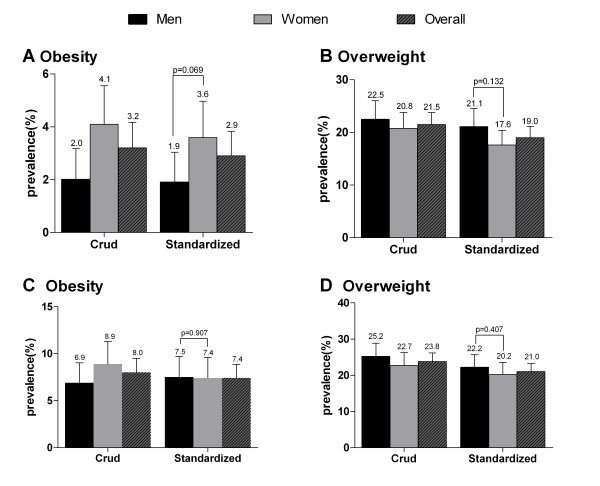
**Crude and age-standardized prevalence of obesity and overweight in the Yi nationality according to WHO criteria (panels **a **and **b**) and the Working Group on Obesity in China (panels **c **and **d**)**. Bars indicate 95% confidence intervals.

According to WHO definitions, the age- and sex-adjusted prevalence of obesity and overweight was 2.9% and 19.0%, respectively, while it was 7.4% and 21.0%, respectively, based on the Working Group on Obesity in China. Regardless of the definition, there was no difference in the prevalence of obesity and overweight between male and female participants (Figure 1).

**Figure 2:**
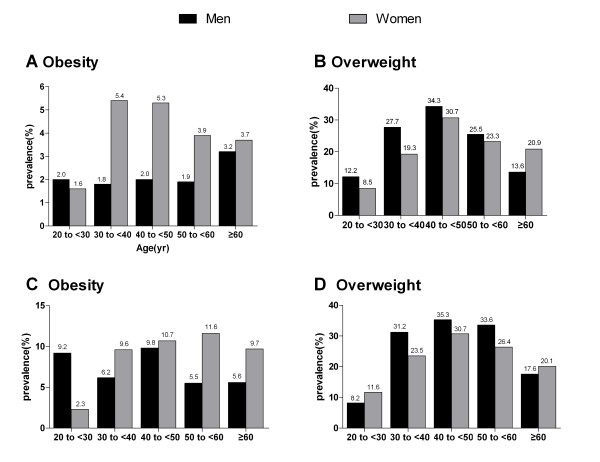
**Sex- and age-stratified prevalence of obesity and overweight in the Yi nationality according to WHO criteria (panels **a **and **b**) and the Working Group on Obesity in China (panels **c **and **d**)**.

According to WHO definitions, men reached their highest prevalence of obesity at ≥ 60 years of age, whereas women had the highest prevalence at 30 to 50 years (Figure 2). If we used China criteria, however, the highest prevalence of obesity was at 40 to 50 years for men and at 50 to 60 years for women. The highest prevalence of overweight was at 40 to 50 years in both men and women, irrespective of criteria (Figure 2).

**Figure 3:**
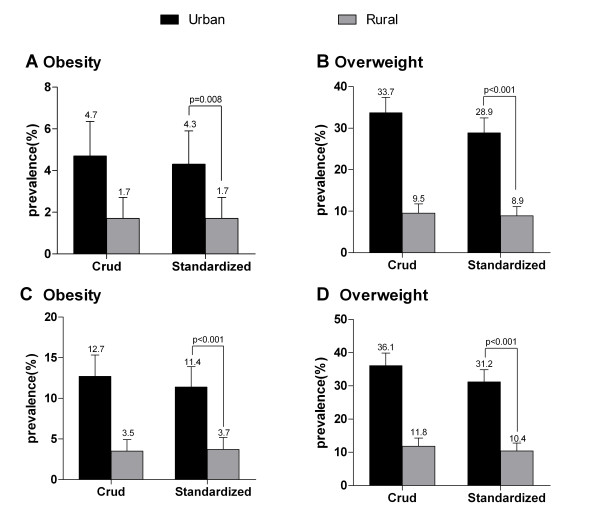
**Crude and age-standardized prevalence of obesity and overweight among urban and rural residents of Yi nationality according to WHO criteria (panels **a **and **b**) and the Working Group on Obesity in China (panels **c **and **d**)**. Bars indicate 95% confidence intervals.

As shown in Figure 3, urban residents had a strikingly higher prevalence of obesity (WHO criteria: 4.3% vs 1.7% *p *= 0.008; China criteria: 11.4% vs 3.7% *p *< 0.001) and overweight (WHO criteria: 28.9% vs 8.9% *p *< 0.001; China criteria: 31.2% vs 10.4% *p *< 0.001) than that in rural residents (Figure 3).

In multivariable logistic regressions, older age, a family history of obesity, higher annual income, drinking and urban residence were significantly associated with an increased risk of overweight/obesity (Table 3).

**Table 3:** Multiple logistic regression for covariate factors and their associations with overweight/obesity

Variable	Overweight/obesity (BMI ≥ 25.0 kg/m^2^)		Overweight/obesity (BMI ≥ 24.0 kg/m^2^)	
	
	OR (95% CI)	P Value	OR (95% CI)	P Value
Age	1.02(1.01-1.03)	0.003	1.02(1.01-1.03)	0.001

Family history of obesity	2.19(2.07-2.32)	< 0.001	2.38(1.56-2.62)	< 0.001

Family annual income				

< 10000 yuan	1(ref)		1(ref)	

≥ 10000 yuan	1.61(1.11-2.33)	0.012	1.86(1.30-2.65)	0.001

Rural residents	1(ref)		1(ref)	

Urban residents	2.82(1.86-4.27)	< 0.001	2.74(1.87-4.01)	< 0.001

drinker	1.39(0.99-1.95)	0.06	1.50(1.09-2.07)	0.013

Male	1(ref)		1(ref)	

Female	1.17(0.79-1.74)	0.42	1.04(0.72-1.51)	0.823

Education (%, n)				

Primary school or below	1(ref)		1(ref)	

Middle school	1.26(0.84-1.90)	0.630	1.16(0.79-1.72)	0.595

College or above	1.13(0.68-1.90)	0.270	1.14(0.70-1.87)	0.446

Never smoker	1(ref)		1(ref)	

Ex-smoker	1.30(0.71-2.35)	0.40	0.82(0.46-1.48)	0.515

Current smoker	0.96(0.64-1.44)	0.84	0.85(0.58-1.25)	0.404

Regular leisure-time physical activity	1.03(0.73-1.46)	0.85	1.25(0.90-1.73)	0.183

CI: confidence interval; OR: odds ratios; BMI: body mass index

## Discussion

This study showed, for the first time, the prevalence of overweight and obesity in a relatively large population of Liangshan Yi ethnic minority, out of a total population of 2.31 million. In our study population, the overall prevalence of overweight and obesity was 19.0% and 2.9%, respectively, based on the WHO definition, while it was 21.0% and 7.4% according to the Working Group on Obesity in China, which is similar to findings reported in the 2002 Chinese National Nutrition and Health Survey (WHO criteria: 18.9% and 2.9%; China criteria: 22.8% and 7.1% among adults aged ≥ 18 years) [10]. According to the WHO definition, the prevalence of obesity among men in Yi people is 1.9%, while that among women is 3.6%. Yi people in China exhibit a strikingly lower prevalence of overweight and obesity than that observed in populations of Western countries [10-12]. Among US adults aged ≥ 20 years, data from the National Health and Nutrition Examination Survey data (NHANES) obtained in 2007-2008 showed that the age-adjusted prevalence of obesity was 33.8% and the corresponding prevalence estimate for overweight and obesity combined was 68.0% [13]. In Europe, a report by Berghöfer et al. [14] showed that the lowest prevalence of obesity in men was no higher than 4.0% and in women it was no higher than 6.2% before 2003. Additionally, Klumbiene et al. [15] reported that by 1998, the prevalence of obesity among men and women was 10% and 15% in Estonia, 11% and 10% in Finland, and 10% and 18% in Lithuania, respectively.

The prevalence of overweight and obesity varies by region or by ethnic group between men and women. The prevalence of obesity among women is higher than that among men in the Iranian population [14]. In Japan, obesity is more prevalent in men than in women [15]. A study by Chen showed that men have a much higher prevalence of overweight and obesity than women in Fujian province of China [16]. In our study, women tended to have a slightly higher prevalence of obesity than men based on the WHO definition of obesity (3.6% vs 1.9%, *p *= 0.069). However, according to the criteria of the Working Group on Obesity in China, there were no significant differences in the prevalence of obesity between men and women. The prevalence of overweight among men was comparable with that among women regardless of criteria. Likewise, sex was also not associated with the risk of overweight/obesity in multivariate analysis. Similar to the findings of our study, the prevalence of overweight/obesity in 3 minority nationalities from the pasture area of Xinjiang in China was found to be comparable between women and men (48.5% vs. 47.5%, *p *= 0.65) [17]. The reason for the variability in the prevalence of obesity between men and women in different countries or regions is not well understood. Differences in lifestyle and sociodemographic variables, as well as other genetic or behavioral factors could explain the observed sex differences.

Along with economic growth and the urbanization of lifestyle in China, the prevalence of overweight/obesity among both urban and rural residents has been on the rise. However, rural people have had a greater increment in the prevalence of diseases than that of their urban counterparts. The disparity in the prevalence of overweight/obesity between urban and rural areas is narrowing. For example, the prevalence of overweight/obesity (a BMI of ≥ 24.0) in the general Chinese population was 26.0% for urban residents and 11.8% for rural residents in 1992. By 2002, the prevalence was 31.6% for urban residents and 20.0% for rural residents. This urban-rural disparity has considerably diminished since 2002 [18]. Similar trends have also been found in the prevalence of hypertension [19] and diabetes [20], which are often known as obesity related diseases. In our study, however, the prevalence of overweight/obesity (a BMI of ≥ 24.0) in urban residents was 3 times as much as that in rural residents (42.6% vs 14.1%). The prevalence of overweight/obesity in urban residents was slightly higher than that of rural residents among the general Chinese population in 1992, whereas rural residents had a strikingly higher prevalence than that in urban residents among Chinese people in 2002. Data from the International Collaborative Study of Cardiovascular Disease in Asia (InterASIA) showed that the prevalence of a BMI of ≥ 25.0 in urban residents was only 47% higher than that in rural residents (39.1% vs. 26.6%, respectively) among Chinese adults aged 35 to 74 years [21]. In the present study, urban residents also had a 3 times higher prevalence of a BMI of ≥ 25.0 compared with their rural counterparts (33.2% vs 10.6%) among the Yi nationality. These survey results indicated that urban Yi residents have become an emerging population who has to face an epidemic public health problem and deserve more attention in health education such as lifestyle modification. In multivariate analysis, adjusting for other potential risk factors, a higher annual income and urban residence were independently associated with an increased risk of overweight/obesity. With economic growth and increased family income in Yi Autonomous Prefecture, Yi nationality in urban areas consume an increasing amount of energy-dense foods, which have undoubtedly contributed to the increase in BW. The rural residents, however, present with a lower prevalence of overweight and obesity, which may be relevant to the remarkably low household income and preserving distinctive diet habits in which coarse grains rather than energy-dense foods are staples [22]. In the current study, urban inhabitants were more likely to participate in leisure-time physical activity. However, there was no significant correlation between leisure-time physical activity and a lower risk of overweight/obesity in multivariate analysis. This may be because urban residents have less energy expenditure in daily life and work even though they engage in more leisure-time physical activity. Leisure-time physical activity was the main method of increased energy expenditure in urban residents, whereas rural residents have to earn their living from working on a farm. In actual life, energy expenditure of rural residents is usually higher than that of urban residents, which may partially explain the geographic difference [2]. Unfortunately, we failed to assess the intensity of labor on the farm. Additionally, our study also confirmed the conventional risk factors for overweight/obesity - age and a family history of obesity- in the minority population. It is well understood that the aging process affects obesity prevalence. Obesity is known to be a more prominent problem at older ages since physical exertion reduces with age and social and behavioral patterns lead to gain weight [23]. In accordance with the ATTICA study [24], our results also showed that overweight and obese participants were more likely to consume higher quantities of alcoholic beverages compared with those of normal weight. In the current study, we found that people with lower levels of education did not have an increased risk of overweight/obesity. In the ATTICA study, however, obese participants were less educated [24], which has also been supported by other studies in the United States [25]. Although the reason for the difference between our study and the others is not clear, it may be due to differences in income and dietary pattern.

The findings in this report are subject to at least three limitations. First, our study was a cross-sectional design, and therefore, cannot establish causal relations. Nevertheless, the findings provide important demographic insights into the growing problem of obesity in Chinese Yi nationality. Further research is required in prospective studies to determine the underlying lifestyle and dietary patterns contributing to this problem. Second, the response rate was higher in women than that in men, which may have resulted in a mild deviation from the exact prevalence of overweight and obesity. Third, we could not measure total physical activity with our data, nor could we determine causal relationships behind BW and energy intake as well as energy consumption.

## Conclusions

Among the Liangshan Yi ethnic minority in China, the total prevalence of overweight and obesity is almost equivalent to that of China 5 years previously. However, urban residents have a much higher prevalence of overweight and obesity than that in rural counterparts. Urban residents have become an emerging population who has to face an epidemic public health problem and deserve more attention in health education such as lifestyle modifications.

## Competing interests

The authors declare that they have no competing interests.

## Authors' contributions

YG performed the statistical analysis and prepared the manuscript. HMT was responsible for the study design and coordination, guided the statistical analysis and revised the manuscript. XWR and RY were responsible for the study design and coordination and reviewed the manuscript critically. YG, XHX, HLL, TC and YL collected the data and reviewed the manuscript. All authors read and approved the final manuscript.

## Pre-publication history

The pre-publication history for this paper can be accessed here:

http://www.biomedcentral.com/1471-2458/11/919/prepub

## Supplementary Material

Additional file 1 **Brief introduction of Liangshan Yi Autonomous Prefecture and Chinese Yi nationality**. Click here for file
