# Supplementary material for: Prevalence of overweight and obesity among Chinese Yi nationality: a cross-sectional study
Source: BMC Public Health. 2011 Dec 13;11:919. doi: 10.1186/1471-2458-11-919 (PMC3272001; doi:10.1186/1471-2458-11-919)
Supplement: Additional file 1 — Brief introduction of Liangshan Yi Autonomous Prefecture and Chinese Yi nationality. [file 1471-2458-11-919-S1.DOC]

**Brief introduction of Liangshan Yi Autonomous Prefecture and Chinese Yi nationality**

Liangshan Yi Autonomous Prefecture locates in the southwest of Sichuan province with south to the Jinsha River, north to the Dadu River, east to the Sichuan Basin and west linking Hengduan Mountain, between 100°15'-103°53'E and 26°03'- 29°27'N. It governs 17 counties (cities) and has an area of 6.04 million M^2^ and a population of 4.73 million including 2.31 million of Yi minority people which acounts for 48.9% of the total and makes it the largest Yi community. Xichang, the state capital, is the centre of the economy, politics and culture. It is a city where spring stays and the world-famous aerospace city.

**History backgrounds**

Socio-economic development in the Yi areas was lopsided before liberation, due to oppression and exploitation by the reactionary ruling class, as well as historical and geographical differences. The socio-economic structure fell by and large into two types – feudalism and slavery. Most of the Yis in Yunnan, Guizhou and Guangxi had entered feudal society earlier on, and a developed landlord economy had emerged in most areas except for remnants of the manorial economy in some areas of northeastern Yunnan and northwestern Guizhou. Slavery remained intact for a long time in the Greater Liangshan Mountain area in Sichuan and the Lesser Liangshan Mountain area in Yunnan.

Slavery kept production at an extremely low level for a long time in the Greater and Lesser Liangshan Mountain areas in Sichuan and Yunnan. While agriculture was the main line of production, land lay waste and production declined strikingly. Slash-and-burn cultivation was still practiced in some mountain areas. The lack of irrigation facilities and adequate manure, coupled with heavy soil erosion, lowered average grain output to less than a ton per hectare. Animal husbandry was a major sideline with sheep making up a large part of the livestock.

For many centuries, barter was the form of trading among the Yis in the Liangshan Mountain areas. Goods for exchange mainly included livestock and grain. Salt, cloth, hardware, needles and threads and other daily necessities were available only in places where Yis and Hans lived together. Occasionally, some Han merchants, guaranteed safe-conduct by Yi headmen, carried goods into the Liangshan Mountain areas.

Due to complex historical reasons, the slave system of the Yis in the Liangshan Mountains lasted till 1949. Before 1949, the Yis in the Liangshan Mountain areas were stratified into four different ranks – "Nuohuo," "Qunuo," "Ajia" and "Xiaxi." The demarcation between the masters and the slaves was insurmountable. The rank of "Nuohuo" was determined by blood lineage and remained permanent, the other ranks could never move up to the position of rulers. "Nuohuo," meaning "black Yi," was the highest rank of society. Being the slave-owning class, Nuohuo made up 7 percent of the total population. "Qunuo," meaning "white Yi," was the highest rank of the ruled and made up 50 percent of the population. This rank was an appendage to the black Yis personally and, as subjects under the slave system, they enjoyed relative independence economically and could control "Ajia" and "Xiaxi" who were inferior to them. "Ajia" made up one third of the population, being rigidly bound to black Yi or Qunuo slave owners, who could freely sell, buy and kill them. "Xiaxi" was the lowest rank, accounting for 10 percent of the population. They had no property, personal rights or freedom, and were regarded as "talking tools."

The founding of the People's Republic of China in 1949 ended the bitter history of enslavement and oppression of the Yis and people of other nationalities in China. The Liangshan Yi Autonomous Prefecture of Sichuan was established in 1952. In the spring of 1958, democratic reforms concluded in the Yi areas in the Greater and Lesser Liangshan Mountains in Sichuan and Yunnan. The reforms destroyed slavery, abolished all privileges of the slave owners, confiscated or requisitioned land, cattle, farm tools, houses and grain from the slave owners, and distributed them among the slaves and other poor people. The democratic reforms inspired the emancipated slaves and poor peasants to reshape their land and expand agricultural production steadily.

**Traditional customs**

In most Yi areas, maize, buckwheat, oat and potato were staples. Rice production was limited. Most poor Yi peasants lived on acorns, banana roots, celery, flowers and wild herbs all the year round. Salt was scarce. In the Yi areas, potatoes cooked in plain water, pickled leaf soup, buckwheat bread and cornmeal were considered good foods, which only the well-to-to Yis could afford. At festivals, boiled meat with salt was the best food, which only slave owners could enjoy.

Cooking utensils of a distinct ethnic color, made of wood or leather, have been preserved in some of the Yi areas. Tubs, plates, bowls and cups, hollowed out of blocks of wood, are painted in three colors – black, red and yellow – inside and outside, and with patterns of thunderclouds, water waves, bull eyes and horse teeth. Wine cups are hollowed out of horns or hoofs.

Yi costume is great in variety, with different designs for different places. In the Liangshan Mountains, men wear black jackets with tight sleeves and right-side askew fronts, and pleated wide-bottomed trousers. Men in some other areas wear tight-bottomed trousers. They grow a small patch of hair three or four inches long on the pate, and wear a turban made of a long piece of bluish cloth. The end of the cloth is tied into the shape of a thin, long awl jutting out from the right-hand side of the forehead. They also wear on the left ear a big yellow and red pearl with a pendant of red silk thread. Beardless men are considered handsome. Women wear laced or embroidered jackets and pleated long skirts hemmed with colorful multi-layer laces. Black Yi women used to wear long skirts reaching to the ground, and women of other social ranks wore skirts reaching only to the knee. Some women wear black turbans, while middle-aged and young women prefer embroidered square kerchiefs with the front covering the forehead like a rim. They also wear earrings and like to pin silver flowers on the collar. Men and women, when going outdoors, wear a kind of dark cape made of wool and hemmed with long tassels reaching to the knee. In wintertime, they lined their capes with felt.

**Economic development and dietary structures since 1978 (the Chinese Economic Reform)**

During the last three decades, Liangshan holds on the policy of advantage resources exploitation and protection to strengthen and thrive the Prefecture by developing the industrial，ecology and opening up. The whole area has grown at an accelerated rate and people's livelihood has improved constantly. By the end of 2006， the prefecture GDP reached 35.96 billion yuan. Annual per capita disposable income for urban people hit 8423 yuan while annual per capita net income for rural households stood at 2717 yuan. However, the gap between urban and rural household incomes is still large. Our survey results showed that the percentage of urban residents who had family annual income of ≥10000 yuan was 58.8% but the percentage was only 5.8% in their rural counterparts.

It is universally accepted that family income is an important factor to affect dietary nutrients intake and dietary structure. In 2003, Zhou Jichang et al. reported that, like the Chinese Han population, urban residents in Chinese Yi nationality have been enjoying a much richer, more diverse diet. There is a trend toward increasing reliance on animal products relative to vegetal products for dietary energy. Their main cooking techniques are stir-frying, deep-frying and steaming. Most of residents in rural areas, however, still retain their traditional lifestyle and dietary structure. The main features are as follows: (1) Vegetal products are predominant dietary patterns. Maize, buckwheat, oat and potato were their staples. (2) Steaming and boiling are the main cooking methods. (3) A narrow variety of foods is available for rural inhabitants. Per capita consumption of animal-source food remains low and is far beyond the Recommended Nutrient Intakes.

**References**

1. <http://www.lsz.gov.cn/tzzn-en>

2. http://www.china.org.cn/english/features/EthnicGroups/136960.htm

3. <http://www.tjcn.org/tjgb/201001/2862_3.html>

4. Zhou J, Huang C, Xu Y, Sun G, Li X, *et al*: **The dietary patterns and nutritional status of adult Yi people in Liangshan autonomous region [in Chinese].** *Wei Sheng Yan Jiu* 2003,32:246-8.


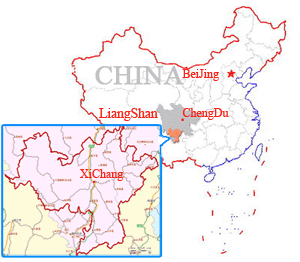


Fig.1 Location of Liangshan Yi Autonomous Prefecture


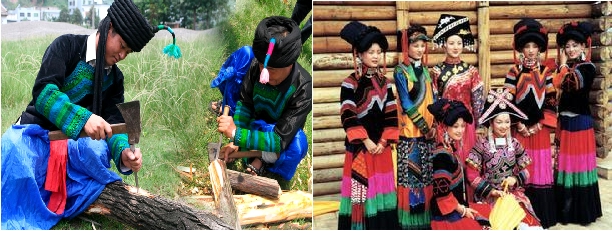


Fig.2 Yi ethnic minority costume


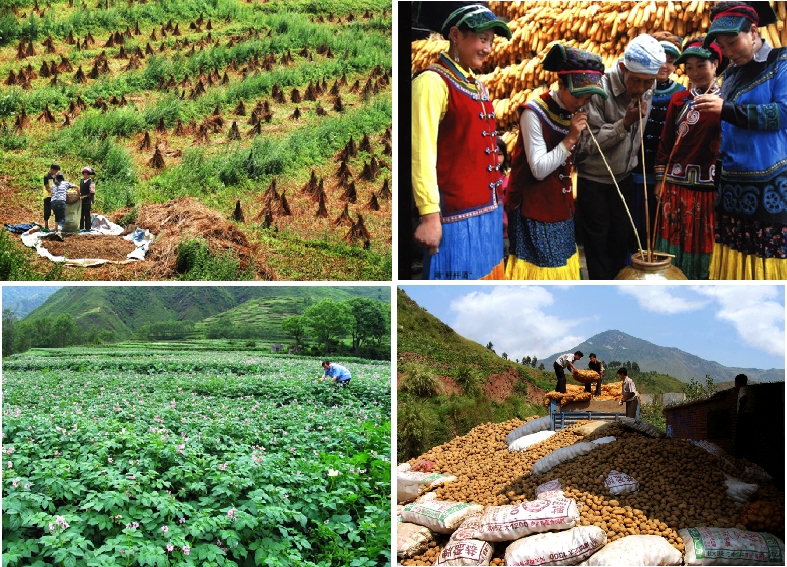


Fig.3 Major farm crops including buckwheat, maize, and potato in Liangshan Yi Autonomous Prefecture
